# Neuropsychological evaluation and rehabilitation in multiple sclerosis (NEuRoMS): protocol for a mixed-methods, multicentre feasibility randomised controlled trial

**DOI:** 10.1186/s40814-022-01073-5

**Published:** 2022-06-11

**Authors:** Gogem Topcu, Laura Smith, Jacqueline R. Mhizha-Murira, Nia Goulden, Zoë Hoare, Avril Drummond, Deborah Fitzsimmons, Nikos Evangelou, Klaus Schmierer, Emma C. Tallantyre, Paul Leighton, Kimberley Allen-Philbey, Andrea Stennett, Paul Bradley, Clare Bale, James Turton, Roshan das Nair, On behalf of the NEuRoMS Collective

**Affiliations:** 1 Mental Health and Clinical Neurosciences, School of Medicine, University of Nottingham, Nottingham, UK; 2 School of Psychology, Keynes College, University of Kent, Canterbury, Kent, UK; 3 North Wales Organisation for Randomised Trials in Health Clinical Trials Unit, Bangor University, Bangor, UK; 4 School of Health Sciences, University of Nottingham, Nottingham, UK; 5 Swansea Centre for Health Economics, School of Health and Social Care, Faculty of Medicine, Health and Life Sciences, Swansea University, Swansea, UK; 6 Department of Neurology, Nottingham University Hospitals NHS Trust, Nottingham, UK; 7 Centre for Neuroscience, Surgery and Trauma, Barts and the London School of Medicine and Dentistry, The Blizard Institute, Queen Mary University of London, London, UK; 8 Clinical Board Medicine (Neuroscience), The Royal London Hospital, Barts Health NHS Trust, London, UK; 9 Division of Psychological Medicine and Clinical Neurosciences, School of Medicine, Cardiff University, Cardiff, UK; 10 Department of Neurology, Cardiff and Vale University Health Board, Cardiff, UK; 11 Centre of Evidence Based Dermatology, School of Medicine, University of Nottingham, Nottingham, UK; 12 Wolfson Institute of Population Health, Queen Mary University of London, London, UK; 13 Multiple Sclerosis Patient and Public Involvement Group, Nottingham, UK; 14 Institute of Mental Health, Nottinghamshire Healthcare NHS Foundation Trust, Nottingham, UK

**Keywords:** Multiple sclerosis, Cognition, Cognitive screening, Rehabilitation, Feasibility study, Randomised controlled trial

## Abstract

**Background:**

Cognitive problems affect up to 70% of people with multiple sclerosis (MS), which can negatively impact mood, ability to work, and quality of life. Addressing cognitive problems is a top 10 research priority for people with MS. Our ongoing research has systematically developed a cognitive screening and management pathway (NEuRoMS) tailored for people with MS, involving a brief cognitive evaluation and rehabilitation intervention. The present study aims to assess the feasibility of delivering the pathway and will inform the design of a definitive randomised controlled trial (RCT) to investigate the clinical and cost-effectiveness of the intervention and eventually guide its clinical implementation.

**Methods:**

The feasibility study is in three parts. Part 1 involves an observational study of those who receive screening and support for cognitive problems, using routinely collected clinical data. Part 2 is a two-arm, parallel group, multicentre, feasibility RCT with a nested fidelity evaluation. This part will evaluate the feasibility of undertaking a definitive trial comparing the NEuRoMS intervention plus usual care to usual care only, amongst people with MS with mild cognitive problems (*n* = 60). In part 3, semi-structured interviews will be undertaken with participants from part 2 (*n* = 25), clinicians (*n* = 9), and intervention providers (*n* = 3) involved in delivering the NEuRoMS cognitive screening and management pathway. MS participants will be recruited from outpatient clinics at three UK National Health Service hospitals.

**Discussion:**

Timely screening and effective management of cognitive problems in MS are urgently needed due to the detrimental consequences of cognitive problems on people with MS, the healthcare system, and wider society. The NEuRoMS intervention is based on previous and extant literature and has been co-constructed with relevant stakeholders. If effective, the NEuRoMS pathway will facilitate timely identification and management of cognitive problems in people with MS.

**Trial registration:**

ISRCTN11203922. Prospectively registered on 09.02.2021.

**Supplementary Information:**

The online version contains supplementary material available at 10.1186/s40814-022-01073-5.

## Background

Multiple sclerosis (MS) is a progressive condition, often diagnosed in young adulthood or early middle age, and is a leading cause of neurological disability in young adults [1]. Up to 70% of people with MS experience cognitive problems [2], which can impact adversely on quality of life, daily activities, and employment [3–6]. Cognitive deficits can manifest as an inability to pay attention, forgetfulness, and problem-solving difficulties. The economic impact of cognitive impairment in MS is felt by the people with MS, their families, and wider society [7] and is likely to escalate with more people being diagnosed and with lower mortality rates. A national survey found cognitive problems were the most debilitating and distressing MS symptoms [8], so addressing cognitive problems, unsurprisingly, is identified as a James Lind Alliance/MS Society ‘top 10’ research priority for people with MS [9].

For people with MS, neuropsychological management (e.g. psychoeducation, internal and external compensatory strategies) can ameliorate the effects of cognitive problems (e.g. loss of independence) and improve mood, employment prospects, quality of life, activities of daily living, and DMT adherence [3, 10, 11]. Clinicians’ and patients’ knowledge of cognitive screening results can also facilitate shared decision-making regarding dose or choice of MS disease-modifying therapies (DMTs).

In the UK, almost 45% of MS specialists report that existing neuropsychological services are insufficient [12–15]. Currently, most UK MS clinics do not routinely screen for cognitive problems and provide insufficient support for most patients with MS who present with these problems [12–16]. By not intervening early, people with MS are less likely to benefit from rehabilitation [17]. Therefore, there is a need for MS services to identify and triage people with MS for timely cognitive assessment and develop and deliver appropriate, resource-efficient interventions, making full use of technological advances.

This feasibility study is part of a larger programme of research: the neuropsychological evaluation and rehabilitation in multiple sclerosis (NEuRoMS; www.neuroms.org) project [18]. NEuRoMS aims to develop and evaluate a pathway for routine screening of cognitive problems and includes a brief cognitive intervention programme for people with MS with mild cognitive problems. In earlier stages of the project, we systematically designed a blueprint of the cognitive screening and management pathway based on extant literature and stakeholder consultation [19, 20] and refined it through implementing it at three NHS hospitals [21, 22].

Prior to testing the pathway in a definitive randomised controlled trial (RCT), we need to assess key feasibility parameters of delivering the cognitive screening and management pathway in MS. This stage is in accordance with Medical Research Council (MRC) guidelines for development and evaluation of ‘complex interventions’ [23]. We will, therefore, determine these parameters, identify any operational issues in delivering the pathway, develop intervention fidelity tools, and produce a framework for cost-effectiveness analyses. We will also examine ‘usual care’ further (adding to data from our earlier work packages [19, 20]) and explore the potential for contamination when delivering the intervention. In addition, the logic model and underlying programme theory [20] will be further developed to move incrementally towards a well-established pathway founded on explicit application to real-world clinical settings.

## Methods

### Aim and objectives

The aim is to assess the feasibility of conducting a definitive RCT to investigate the clinical and cost-effectiveness of the NEuRoMS intervention in reducing the impact of cognitive problems in daily life amongst people with MS and the acceptability of the intervention.

The specific objectives, mapped onto different parts of the study, are listed in Table 1.

**Table 1:** List of specific objectives, mapped onto different parts of the study

No.	Objectives
Part 1 — Testing cognitive screening pathway	
1.	Explore how the NEuRoMS cognitive screening and management pathway is integrated within routine clinical practice
2.	Refine the cognitive screening pathway by evaluating online cognitive screening and usage data and the observations of clinicians/intervention providers
3.	Assess suitability of online cognitive screening tool for capturing cognitive deficits
4.	Assess the frequency and extent of no, mild, and moderate-severe cognitive deficits and, thus, the size of the target population (potentially eligible participants for a future definitive RCT) based on online cognitive screening tool
Part 2 — Acceptability, feasibility RCT, and fidelity evaluation	
1.	Identify the necessary parameters and tools to undertake a clinical and cost-effectiveness analysis in a future definitive trial
2.	Assess acceptability of data collection tools, processes, data completeness, and follow-up rates and determine suitability of outcome measures
3.	Identify factors that may affect running of the definitive trial, including barriers and facilitators to recruitment, retention, and delivery of the intervention
4.	Evaluate the feasibility and acceptability of the NEuRoMS intervention
5.	Evaluate and optimise intervention usage and acceptability
6.	Explore ways to assess (type and extent) and minimise contamination
7.	Develop and assess intervention fidelity tools
8.	Develop a framework for cost-effectiveness analyses
9.	Characterise ‘usual care’ in the different sites
Part 3 — Exploring stakeholder views and experiences	
1.	Gather detailed qualitative feedback interviews on the pathway, intervention, and study procedures to assess their feasibility and acceptability
2.	Understand the barriers, facilitators, and broader context for delivering and receiving screening and management pathway
3.	Improve understanding of how the NEuRoMS screening and management pathway is integrated within routine clinical practice
4.	Improve understanding of how the NEuRoMS intervention programme is experienced by those who deliver and receive it
5.	Evaluate and refine staff training package for cognitive screening and management pathway
6.	Refine the programme theory (and logic model) for the newly developed screening pathway and NEuRoMS intervention programme, embedding it in clinical practice

### Study design

**Fig. 1:**
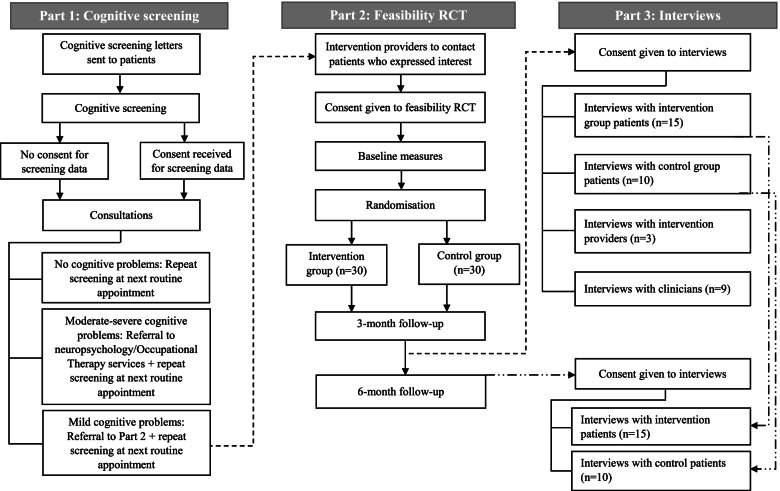
Study flowchart

This is a multicentre mixed-methods feasibility study and fidelity evaluation. The study is in three parts. Part 1 is an observational study of those who receive screening and support for cognitive problems, using routinely collected clinical data. In part 2, we will evaluate the feasibility of undertaking a definitive trial comparing NEuRoMS intervention plus usual care to usual care only, amongst people with MS with mild cognitive problems, using a parallel group, multicentre, feasibility RCT with a nested fidelity evaluation. In part 3, semi-structured interviews will be undertaken with participants in the feasibility RCT, and clinicians and intervention providers involved in delivering the NEuRoMS cognitive screening and management pathway. A SPIRIT figure (Table 2) and study flowchart (Fig. 1) outline the study flow; a completed SPIRIT checklist is available as an additional file.

**Table 2:**
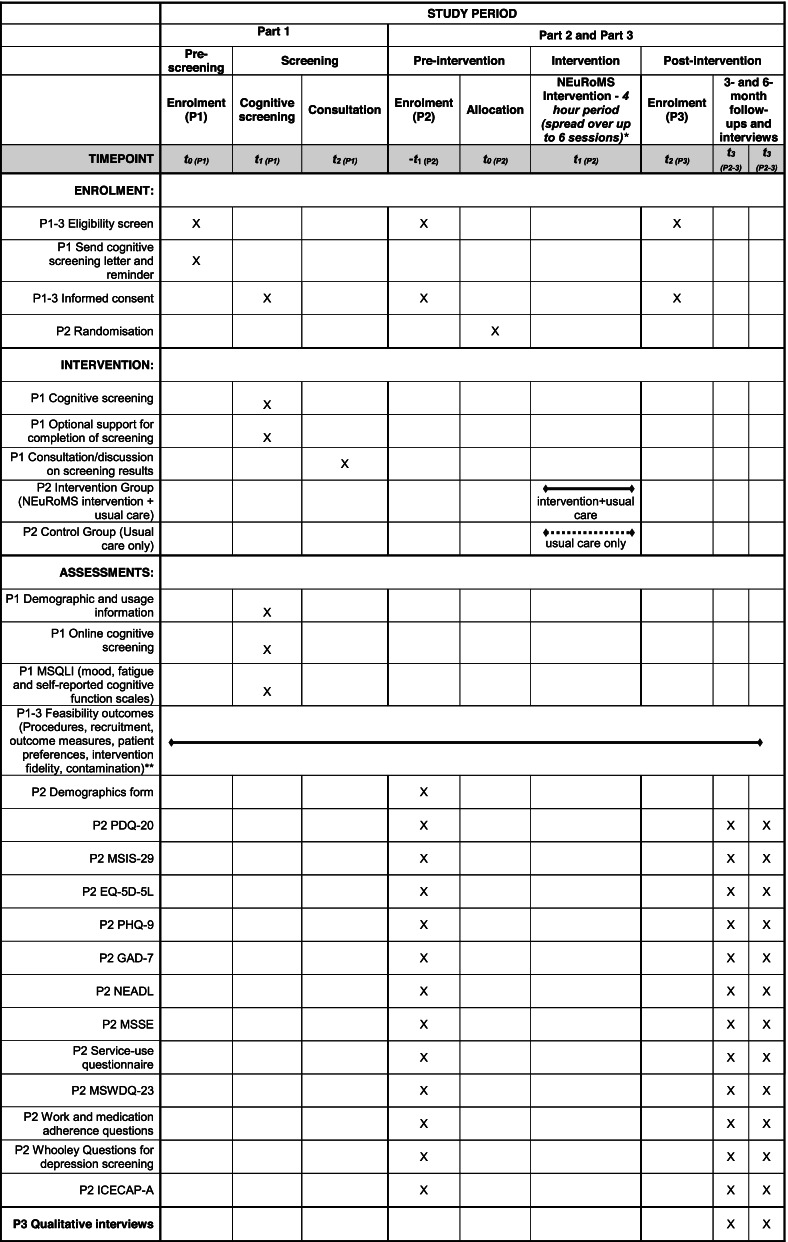
SPIRIT figure — schedule of enrolment, interventions, and assessments for parts 1, 2, and 3

*The time between sessions will be variable, in keeping with participants’ schedules and other commitments. **The key outcomes of interest in this study relate to the feasibility of proceeding to a larger definitive trial. Feasibility will be measured throughout the data collection period. Key: *P1*, part 1; *P2*, part 2; *P3*, part 3; *t*, timepoint; *GAD-7*, General Anxiety Disorder-7; *ICECAP-A*, ICEpop CAPability measure for Adults; *MSIS-29*, Multiple Sclerosis Impact Scale-29; *MSQLI*, Multiple Sclerosis Quality of Life Inventory; *MSSE*, Multiple Sclerosis Self-efficacy Scale; *MSWDQ-23*, Multiple Sclerosis Work Difficulties Questionnaire-23; *NEADL*, Nottingham Extended Activities of Daily Living Scale; *PDQ-20*, Perceived Deficits Questionnaire; *PHQ-9*, Patient Health Questionnaire-9; *SST*: symbol substitution test; *WCT*, word colour test

### Participating centres

Participants will be recruited from MS outpatient clinics within three UK NHS hospitals: (1) Barts Health NHS Trust, (2) Cardiff and Vale University Health Board, and (3) Nottingham University Hospitals NHS Trust.

### Study population

Participants for the feasibility study comprise people with MS, intervention providers, and clinicians.

#### Inclusion criteria

In part 1, we will include people identified by the clinical team as having a diagnosis of MS. In part 2, people with MS will be considered eligible if they completed cognitive screening (part 1) and were identified as having mild cognitive problems using pre-determined cut-offs [24, 25] and data from previous work [21]. In part 3, individual participants to be included should meet the following criteria: (1) people with MS who participated in part 2 feasibility RCT, (2) intervention providers (e.g., assistant psychologists/research nurses/assistant occupational therapists) delivering the NEuRoMS intervention to people with MS in part 2 feasibility RCT, and (3) clinicians (e.g., neurologists, MS nurse specialists, psychologists, occupational therapists) delivering the NEuRoMS screening and management pathway to people with MS.

All participants are required to be (1) aged 18 years or above, (2) able and willing to give consent, and (3) able to communicate in English.

#### Exclusion criteria

In part 2, participants will be excluded if they (1) are currently receiving neuropsychological intervention for cognitive problems and (2) previously received NEuRoMS intervention during a previous study [22].

### Participant identification and consent procedures

All patient participants will be initially approached by their clinical team. Study sites will have an intervention provider working at their hospital, who will form part of the MS clinical care team and will assist with participant identification. All participants will provide informed consent before they enter the study.

In part 1, patients at the study sites will be approached by their clinical team ahead of their routine appointment at the MS clinic to complete the online screening. Contact will typically be initiated approximately 1 month before the scheduled appointment but may vary based on how appointments are arranged at each site. Patients will receive a weblink (by post, text message, or email) requesting them to access this link to complete the online cognitive screening at home (with telephone support from the site if required) or in-person in the clinic. Consent to use their screening data in part 1 will be obtained online from patients.

At the scheduled clinic appointment, eligible participants identified as having mild cognitive problems in part 1 will be invited by a member of the clinical care team to participate in the part 2 feasibility RCT following discussions regarding their cognitive screening results. Those agreeing to discuss the study with the intervention providers or research team will be contacted by phone, letter, or email to introduce the study, assess eligibility, answer questions, provide a copy of the participant information sheet, and obtain informed consent. Participants will receive a gift voucher for completing the questionnaires at each follow-up time point (3 months, 6 months) to thank them for their time.

In part 3, patients participating in part 2 who provide consent to be approached by the research team will be invited to part 3 interviews. Clinicians and intervention providers will be approached by the research team. All participants will receive a copy of the participant information sheet and consent form online or by post. Patient participants will also receive a copy of the interview guide. Informed consent will be obtained from all participants via phone or videoconferencing, by post, in-person, or online. Patient participants will receive a gift voucher for participating in interviews at each follow-up time point (3 months, 6 months) to thank them for their time.

### Sample size

Based on reduced screening throughput at each site during the pandemic, and which now persists, we estimate that 930 patients will receive the cognitive screening link as part of their newly introduced clinical care (part 1) in 6 months.

We will recruit 60 participants to the part 2 feasibility RCT. Recommended sample size for feasibility RCTs range between 24 and 50 [26]. We believe 60 participants will enable us to optimally address the aims of this study and provide us with parameter estimates to confirm our sample size calculations for the definitive trial.

In part 3, we will interview 25 patient participants from the feasibility trial (10 from control group and 15 from intervention group) at two time points: after 3-month follow-up (time point no. 1) and after 6-month follow-up (time point no. 2). Additional interviewees will be recruited at time point no. 2 if there are dropouts. We will also interview 9 clinicians (three from each site) and the 3 intervention providers (one from each site). Sample sizes for the interviews are based on extant literature, which suggest these numbers will offer ‘theoretical sufficiency’ to answer specific questions [27] and inform the ongoing development of NEuRoMS. The sample size for the intervention providers is limited because there will only be three providers.

### Randomisation and blinding

In the feasibility RCT, once the participant has been recruited, consented, and completed the baseline questionnaires, we will individually randomise them to control or intervention groups (ratio 1:1, stratified by study site), using an online dynamic adaptive algorithm [28], developed and maintained by the North Wales Organisation for Randomised Trials in Health (NWORTH) Clinical Trials Unit (CTU).

Blinding will not be possible for the participants and intervention providers due to the nature of the intervention. Research staff who are analysing the data will remain blind to allocation. Most outcome data will be self-reported and collected using online or postal questionnaires. Questionnaires collected over the phone will be collected by an independent researcher who will request participants not to reveal their group allocation or discuss their study involvement. At each assessment time point, the independent researcher will be asked to record if they were unblinded to group allocation, and if so, the reasons for this.

### Intervention and usual care groups

All participants will have completed the online cognitive screening process (part 1) pre-randomisation. Cognitive screening is a new clinical procedure at each site involving a self-administered, brief online screening tool (completed at home prior to clinic visit or completed in clinic prior to routine neurology appointment) that can be administered with minimal support from clinical staff. The screening tool consists of computerised tasks (e.g. symbol substitution task which is a version of the Symbol Digit Modalities Test and/or word colour test which is a version of the Stroop test) that capture cognitive functions (information processing and attention) and brief questionnaires, assessing mood, fatigue, and self-reported cognitive function from the Multiple Sclerosis Quality of Life Inventory [29]. Test and questionnaire scores will be automatically calculated by the online system and transferred to the clinical team. This new clinical procedure will form part of routine care and will help identify cognitive problems and facilitate discussions between patients and clinicians, to encourage joint decisions about appropriate management for these problems.

In part 2, the intervention group will receive usual care plus the NEuRoMS cognitive management intervention. The control group will receive usual care only. The NEuRoMS intervention is a therapist-led, manualised, and multi-faceted programme, involving various components (e.g. information provision, goal setting) and a range of strategies and techniques (e.g. psychoeducation, compensatory strategies, boosting cognitive reserve). The intervention is person centred, tailored to the needs and lifestyle of each participant, and aims to help people with MS cope with and manage cognitive problems by establishing strategies that can be maintained once the intervention sessions are completed.

The intervention will be delivered by a trained therapist (i.e. intervention provider), under the supervision of a clinical psychologist or occupational therapist. In-person, videoconferencing, and telephone delivery options will be available. The duration of the intervention will be up to 4 h, spread across up to 6 sessions. We anticipate these sessions to occur over a 2-month period, based on patient availability.

The content of usual care, based on our knowledge of these sites, is our online cognitive screening plus signposting to the MS Society/MS Trust websites. We will document usual care received through a service-use questionnaire, clinical notes, and part 3 interviews.

### Outcomes

The key outcomes of interest relate to the feasibility of proceeding to a definitive trial. The primary endpoints are therefore based on part 2, and the secondary endpoints relate to part 1 and part 3. Table 3 presents the primary endpoints, and Table 4 presents the secondary endpoints, including the method of assessment, assessment timepoints, and the corresponding study objective.

**Table 3:** Primary endpoints based on part 2

Outcome	Assessment procedure	Assessment timepoints	Corresponding study objectives
Feasibility and suitability of trial procedures	Response rates, trial uptake, and number of dropouts	Throughout data collection period	Part 2: objectives 1, 2, and 3
Feasibility of recruitment	Appropriateness of eligibility criteria: number of participants referred or interested who meet the eligibility criteria	Throughout data collection period	Part 2: objectives 1, 2, and 3
	Recruitment rate: number of eligible patients who consent and decline to participate (including reasons for nonparticipation)		
	Retention rates: number of participants who consent and remain in the trial by 6-month follow-up		
Appropriateness of self-report clinical and health economics measures	Completion rates, rate of return, data completeness (the number of missing data), number of reminders and requests for extra support to complete measures, and content of contacts between service providers/researchers and patient participants	Baseline, 3-month and 6-month follow-up	Part 2: objectives 1 and 2
Patient preference for different versions/formats of the outcome data collection tools	Number completed via paper/online/telephone, the number and types of reminders required to complete measures, data completeness, the number of participants who complete 3-month and 6-month follow-up measures	Baseline, 3-month and 6-month follow-up	Part 2: objectives 1 and 2
Fidelity of the intervention	Accuracy and quality of intervention delivery: intervention record forms, audio/video recordings, and case notes of intervention sessions	Throughout data collection period	Part 2: objectives 3, 4, 5, 6, and 7
	Contextual and process issues related to intervention delivery: clinical notes, intervention record forms, audio/video recordings of intervention sessions, and monthly supervision sessions with the NEuRoMS therapists and part 3 interviews		
Documentation of usual care and contamination	Record of any cognitive support provided by the clinical team: review of clinical notes, resource-use questionnaires, and through monthly teleconferences/videoconferences with clinicians, monthly supervision sessions with the NEuRoMS therapists, and the part 3 interviews	Throughout data collection period	Part 2: objectives 8 and 9
	Record of potential sources of contamination in the control group: Review of clinical notes, resource-use questionnaire, and through monthly supervision and mentoring sessions with the NEuRoMS therapists and the Part 3 interviews

**Table 4:** Secondary endpoints based on part 1 and part 3

Outcome	Assessment procedure	Assessment timepoints	Corresponding study objectives
**Part 1 — testing cognitive screening pathway**			
Feasibility of the cognitive screening pathway procedures	Number of patients who complete the screening in different settings (at home, in-clinic)	Throughout part 1 data collection period	Part 1 Objectives 1, 2, and 3
	Number of different devices patients use to complete the screening (e.g. tablet, mobile phone, laptop)		
	Time taken to complete the screening (in minutes)		
	Number of patients who require reminders and extra support (telephone/in-clinic) to complete the screening		
	Number and content of contacts between service providers and patient-participants		
Patient scores from the cognitive screening measures	Symbol substitution task and/or word colour test and three measures from the Multiple Sclerosis Quality of Life Inventory for self-reported cognitive problems, fatigue, and mental health	Measured during screening as part of usual care	Part 1 Objectives 3 and 4
**Part 3 — Exploring stakeholder views and experiences**			
Feasibility and acceptability of trial procedures through qualitative data	Interviews will explore patients’ willingness to be randomised; patients’ views on trial recruitment and retention strategies, preferences, barriers, and facilitators to trial recruitment and retention; importance and acceptability of outcome measures	3-month and 6-month interviews with patients and interviews with intervention providers	Part 3 Objective 1
Operational issues in the delivery of cognitive screening and management pathway	Contextual factors which influence intervention and pathway delivery, including mechanisms which influence its affect and outcomes; behavioural elements of the intervention, essential resources needed, and barriers to screening and intervention delivery Reviewing notes from monthly teleconferences/videoconferences with clinicians and supervision sessions with intervention providers and the part 3 interviews	Throughout data collection (notes) 3-month and 6-month interviews with patients and interviews with intervention providers and clinicians	Part 3 Objectives 2, 3, 4, and 6
Patient, intervention provider and clinician experiences of cognitive screening and management pathway	Engagement with the pathway; mechanisms considered important in determining key outcomes: Reviewing notes from monthly teleconferences/videoconferences with clinicians and supervision sessions with intervention providers and the part 3 interviews	Throughout data collection (notes) 3-month and 6-month interviews with patients and interviews with intervention providers and clinicians	Part 3 Objectives 2, 3, 4, and 6
Intervention provider experiences of training	Interviews to explore intervention providers’ readiness to deliver NEuRoMS intervention following training; potential contamination issues; potential improvements to training: reviewing training feedback forms, supervision sessions with intervention providers, and part 3 interviews with intervention providers	Throughout data collection (notes) Interviews with intervention providers	Part 3 Objectives 5 and 6

In addition to feasibility outcomes, the following measures will also be used to capture information about the part 2 participants at baseline and 3 and 6 months after randomisation:

- Cognitive impairment assessed using Perceived Deficits Questionnaire [30]
- Quality of life assessed using Multiple Sclerosis Impact Scale [31] and EQ-5D-5L [32]
- Mood assessed using Patient Health Questionnaire-9 [33], Generalised Anxiety Disorder-7 scale [34], and Whooley Questions for depression screening [35]
- Functional ability assessed using Nottingham Extended Activities of Daily Living Scale [36]
- Self-efficacy assessed using Multiple Sclerosis Self-efficacy Scale [37]
- Service-use questionnaire, based on a measure used in other MS trials [38] and adapted for use in the NEuRoMS project
- Work-related issues assessed using Multiple Sclerosis Work Difficulties Questionnaire Short Form [39]
- The extent to which work and medication adherence have been impacted by cognitive problems assessed using two single bespoke questions
- Capability and wellbeing for health economics evaluation assessed using the ICEpop CAPability measure for Adults (ICECAP-A) [40].

We chose these measures because they (i) tap the domains of interest identified by our patient and public involvement (PPI) group (ii), were endorsed by our PPI partners, (iii) have good psychometric properties and are routinely applied in MS research, and (iv) are brief and easy to complete.

### Data collection

Part 1 involves an observational study of those who receive cognitive screening and support, using routinely collected screening data. Observations of how people use and interact with screening will include anonymised usage data (e.g. duration, mode of completion, frequency and type of support received to aid completion, and the reminders required to complete screening) and screening scores from the completed screening tasks from those who gave consent.

Monthly teleconferences/videoconferences between a member of the research team and clinicians at the three MS clinics will also be documented with detailed notes recorded by the researcher and used as a data source. These conversations will explore any problems with implementation, possible solutions, and what is working well and why.

In part 2, once the participant has been recruited and consented, they will complete the demographics form and baseline questionnaires either online, over the phone with a member of the research team, or using postal paper copies. Participants will complete the same questionnaires at 3- and 6-month post-randomisation. Independent researchers, blinded to treatment allocation, will support participants who require help completing the questionnaires over the telephone and will contact participants to complete any missing questionnaire data.

We will keep a record of any intervention contamination. Contamination will be minimised by having only specific intervention providers deliver the intervention for the trial. Intervention providers will be specifically trained to deliver the intervention, and only they will have access to the intervention tools via a password-protected website. The intervention providers will only provide care to intervention group participants and not to control group participants. We acknowledge some contamination may occur if people with MS share information with others, but as the intervention is individually tailored and delivered one to one, the potential for contamination at a participant level is small. Therefore, we will assess to what extent all participants in both control and intervention groups received the NEuRoMS intervention by reviewing clinical notes and service-use questionnaire and through monthly supervision and mentoring sessions and part 3 interviews.

In part 3, semi-structured interviews will be undertaken with part 2 participants, clinicians involved in delivering the NEuRoMS cognitive screening and management pathway, and intervention providers. Interviews will explore the cognitive screening and management pathway, components of the intervention, and research processes, to refine the pathway, intervention, and programme theory prior to the definitive trial. Control participants will also be interviewed about their feelings about not being randomised to the intervention group, what we can do to improve return of questionnaires, and what care they received (to characterise usual care and assess any contamination). We will also enquire about participants’ experience of receiving their screening results. To aid participant recall, we will collect qualitative data from patient participants at two time points: after completion of 3-month follow-ups to gather feedback on the intervention and after completion of 6-month follow-ups to gather feedback on outcomes.

Interviews will mostly be conducted over the phone/videoconferencing; however, patient participants can request to be interviewed in-person. Semi-structured interview guides will be sent to patient participants ahead of the interview to foreshadow what the interview will cover. Interviews will be audio recorded and transcribed verbatim by an approved transcription service. Interviewers will keep field notes to capture contextual information. The interviewer will have a good understanding of the screening and intervention, but will not be directly involved in delivering either.

### Adverse events

The occurrence of an adverse event (AE) or serious adverse event (SAE) because of study participation is unlikely since the study involves completing standardised tasks and questionnaires and receiving a manualised non-pharmacological intervention with trained professionals. Indeed, this has been the case in other MS trials of a similar intervention [38]. AEs and SAEs such as hospitalisation, distress, and death, in relation to participating in this study, will be recorded; however, these are likely to be rare events due to the nature of the study. ‘Notable events’ (i.e. any events that are considered out of ordinary) occurring during cognitive screening, or the intervention, will be recorded and monitored by the intervention providers or researchers. Participants will be informed of all research activities prior to participating in the study; thus, there will be no deception.

The results of the screening will be discussed with the patient by a healthcare professional, and, therefore, any concerns about the screening results will be addressed by the clinician. It is possible that talking about cognitive difficulties may sometimes create distress for people with MS. This distress will be dealt with during the intervention sessions or interviews on an individual basis. The intervention will be delivered in a clinical environment by a trained intervention provider who will receive monthly supervision. Therefore, the overall risk has been assessed as minimal.

If a participant is identified as having a high score on the Patient Health Questionnaire-9 and Generalised Anxiety Disorder-7 scale as part of the part 2 patient-reported outcomes (≥ 15 indicative of severe anxiety and depression [41]), we will recommend that they contact their GP to discuss this further. This will not affect their participation in the study, and GPs will already be aware of patients’ involvement in the part 2 study.

If a participant discloses information during the intervention sessions or during interviews indicating that they are at risk of self-harm, or discloses information about intention to harm others, the intervention provider and interviewer will follow the safeguarding policies of their employing organisation or local NHS Trust standard procedures. If the participant has responded to question 9 of the Patient Health Questionnaire-9 (“How often have you been bothered with … thoughts that you would be better off dead, or of hurting yourself”) with answer 3 (“Nearly every day”), when checked by the research team, this will be interpreted as a significant suicide risk and documented as an AE. The researcher will report the suicide risk to the principal investigator (PI) immediately. The PI/person delegated by PI will then deal with the situation as per local NHS Trust standard procedures. AEs and SAEs will be reviewed and monitored by the independent programme steering committee.

The interviews will be conducted by trained researchers who will follow a distress protocol in the event of distress during interviews [42]. Researchers will ensure they give sufficient time to participants for debriefing after the intervention sessions/interviews. Participants will be informed that they can end the study at any point. If the participant no longer wishes to continue and consent is withdrawn, the researcher will debrief the participant and provide support, as needed.

Patient participants will be advised in the participant information sheet to contact their general practitioner (GP) (and/or their occupational health/counselling services for clinician and intervention provider participants) if they feel distressed. Contact information of appropriate sources of support will be provided for patient participants who feel they need further information/support with respect to cognitive problems in MS. Participants and GPs of patient participants will also receive the contact details of the research team for enquiries about the research.

### Fidelity evaluation

To ensure fidelity of delivery of the intervention, we will request intervention providers’ and participants’ consent to record intervention sessions (audio or video recording depending on the platform used and participants’ preference). When this is not possible, intervention providers will keep detailed notes. Intervention providers will also complete an intervention record form during/after each session, detailing the content of the therapeutic interaction. We anticipate 48 observations (up to 12 participants (4 per intervention provider) each receiving approximately 4 sessions) will be sufficient to identify intervention manual deviations and skills that require addressing in training for the definitive RCT trial.

A fidelity checklist will be completed based on the Conceptual Framework for Implementation Fidelity [43] for each case exploring adherence to the content, frequency, duration and coverage, and possible factors that moderate the adherence (e.g. intervention complexity, facilitation strategies, quality of delivery, responsiveness). A sample of data from the intervention record forms, from observations, and from case notes of interventions will be used to complete the checklists to identify where and how the intervention delivery deviated from that described in the intervention manual.

Findings will be used to refine the logic model, intervention documents, and training packages delivered to clinical team members and intervention providers for the clinical pathway, as necessary. We will develop and refine fidelity tools for the definitive trial and codebook for audio/video analysis of intervention delivery, based on work by Borrelli [44].

### Statistical analysis

In part 1, descriptive statistics and frequencies will be computed using STATA software to describe screening usage by calculating the proportions of participants who completed the screening in different settings and devices and the frequency and type of support and reminders provided. The proportions of participants with no cognitive problems, mild cognitive problems, or moderate and severe cognitive problems will be computed using pre-determined cut-offs [24, 25] and data from previous work [21], and assessment of scale cut-offs will be made.

A full statistical and health economics analysis plan for part 2 will be written and agreed by the programme management group and the Programme Steering Committee before data collection is completed. As this is a feasibility RCT, analysis will focus on assessing the feasibility criteria to determine whether to proceed to a definitive trial. Recruitment and retention figures will be calculated. This will include an assessment of the size of the eligible population and those that consent to participate. From this accumulated data, we will check the assumptions of the sample size calculation for the RCT. Statistical analysis plans for the RCT will also be finalised with knowledge of the format of the data collected. We will focus on the traffic light stop/review/go criteria [45, 46] to progress to the definitive trial, particularly on recruitment (go, average 12/month; review, average 6–11/month; stop, average ≤ 5/month) and retention (go, ≥ 80%; review, 50–79%; stop, ≤ 49%).

Descriptive statistics will also be computed to summarise questionnaire data overall and per group. Exploratory analysis will be conducted using the principle of intention to treat. A repeated measures ANOVA will be used to assess changes in the questionnaire measures over time. Adjusted mean differences will be reported to assess differences over time and between groups. Analysis will adjust for stratification variables. All estimates will be presented with 95% confidence intervals.

Data from individual participants and subgroups of participants will also be assessed to determine whether any participant, or subgroup of participants, has a larger or smaller magnitude of changes in the questionnaire measures compared with other participants. Any findings will be considered exploratory and will be considered for incorporation into the design of the definitive RCT.

We will evaluate the use of single work question instead of the full MS Work Difficulties questionnaire [39]. Correlation and reliability analyses will be computed between the total score of the MS Work Difficulties questionnaire and the single question. We will also evaluate the use of the Whooley Questions instead of the full PHQ-9. Correlation analysis will be computed between the total score of the PHQ-9 and the Whooley Questions.

Levels of missing data will be monitored and presented as part of the ongoing data collection, cleaning, and reporting processes. For validated outcome measures where rules are identified for handling missing data, these methods will be implemented. As this is a feasibility study, no further imputation will be applied to the data. Suitability of outcome measures will be determined by the level of completeness. Potential key outcome measures (e.g. Multiple Sclerosis Impact Scale-psychological subscale) for the definitive RCT will be deemed appropriate if minimum success criteria are achieved or if we can identify solutions to overcome any identified issue.

### Qualitative analysis

Qualitative data from part 3 will be analysed using a framework approach [47, 48] using QSR NVivo. Data will be coded by research team members, and the coded data will then be mapped to thematic matrices which reflect resources required for cognitive screening and management pathway to be delivered, contextual factors which influence its delivery, mechanisms which influence its affect, and outcomes which are described by participants. This will enhance our understanding of the key issues for stakeholders in relation to screening, intervention, and trial procedures. It will allow us to explore the behavioural elements of the intervention, essential resources, and barriers to screening and intervention delivery and trial procedures. If required, the framework will be revised to include new concepts introduced during the interviews, with input from PPI team members. Once all the data are mapped onto the framework, tables will be used to summarise each main theme. Interpretation of this data will inform a final revision of the logic model and programme theory and the design of the definitive trial. Intervention documents, training package, and outcome measures will be refined as necessary.

### Health economics evaluation

The study will explore the feasibility of collecting resource/service use information through the use of the service use questionnaire that was successfully used in a previous study [38]. It was informed by other measures in similar populations or service/setting (e.g. review of Database of Instruments for Resource-Use Measurement website [49]), with a primary focus on collecting direct healthcare and personal social service costs (NHS/PSS perspective) and considering whether direct and indirect non-healthcare costs can be collected to inform a societal perspective. We refined the measure (with PPI and clinician input) to ensure that it reflects good practice [50].

Drawing on previous studies [19, 20], we will consider the outcome domains that stakeholders identify as being relevant and which measures best map across these domains. Justification for the choice of measure (e.g. EQ-5D-5L, ICECAP-A) will be supported by literature searches to ensure our approach is consistent within the field. We will assess the usefulness of instruments by examining descriptive statistics (e.g. completion rates, missing items) with exploration of possible ceiling effects. This assessment, in conjunction with the qualitative data obtained, will be used to finalise the economic outcome measures to use in the definitive trial, including any refinement needed. The outcome for the health economic evaluation will be to derive the framework for a full health economic evaluation for the definitive trial.

### Data management

All data will be handled confidentially and will be anonymised or pseudonymised (as appropriate) and be kept separate from information about the identity of the participants and documents with patient identifiable data. Each participant will be assigned a unique identity number for use on case report forms, other study documents, and electronic databases. Electronic data including study databases will be held securely and password protected on secure university networks. Paper records will be held securely, in locked rooms and cabinets. Paper records will be entered onto electronic databases by research team. Accuracy of data entry will be checked by a member of the research team who did not complete the original data entry.

### Governance

The study will be overseen by an independent programme steering committee, with an independent chair, expert clinician and researcher, statistician, and PPI representative. The study sponsor is the Nottinghamshire Healthcare NHS Foundation Trust. The study will be managed and coordinated by the programme management group, which consists of the chief investigator, programme manager, PPI coinvestigators, and other co-applicants and researchers involved with the NEuRoMS project.

### Patient and public involvement

This protocol has been developed in conjunction with our PPI coinvestigators (who have MS) and our local MS-PPI group. Our PPI coinvestigators have been involved with every key stage of the research process from identifying research questions, guiding funding applications, developing and testing the cognitive screening technology and protocol development, and informing study design. All patient-facing material has been co-written with, and approved by, our PPI partners.

We will continue to work closely with our MS PPI group to ensure we produce useful findings that can facilitate the transferability of the screening and management pathway into practice in a range of healthcare settings. Our PPI coinvestigators and our PPI group will continue to be involved with every key stage of the research process (e.g. data collection, analysis, write up, and dissemination) providing us with the real-life experiences of living with MS and what they feel is needed.

### Dissemination

Study findings will be disseminated to scientific audiences, people with MS, policymakers, healthcare networks, relevant organisations, and stakeholders. Study findings will be published in peer-reviewed journals, presented at national and international conferences, and made available to relevant patient groups via a range of dissemination channels. Our PPI coinvestigators will also disseminate research findings to the wider MS community and public through local and national networks (e.g. local support groups, newsletters) and through social media and other media channels. Participants will be provided with a lay summary of the findings (produced in consultation with our PPI coinvestigators and PPI group), and the summary will be made available on the NEuRoMS study website. Authorship on publications will be based on the International Committee of Medical Journal Editors Recommendations for the Conduct, Reporting, Editing, and Publication of Scholarly Work in Medical Journals [51].

## Discussion

The need for timely identification and effective management of cognitive problems in MS has been highlighted by people with MS [19, 20], clinical guidelines [52], and International MS Consortia [53, 54]. This protocol describes a feasibility study delivering a cognitive screening and management pathway to identify, triage, and treat cognitive problems in people with MS. Following MRC guidelines [23], this study will evaluate key feasibility parameters of the pathway before proceeding to a definitive trial.

This feasibility study targets people with MS with mild cognitive problems since their level of impairment means they are more likely to benefit from a brief intervention introduced at an earlier stage in their care. Mild cognitive problems are often missed and are poorly understood, partly because the field of cognition in MS has tended to use dichotomous categorisations of ‘intact’ or ‘impaired’ to interpret cognitive performance [55, 56]. The data obtained from this study will broaden our current knowledge of cognitive problems in MS to give a better understanding of the prevalence of people with MS who experience cognitive problems of varying severity and to develop MS cognitive profiles amongst individual participants and subgroups of participants. This new understanding will help form relevant homogenous target groups for research studies and enable clinical teams to tailor support to patterns of cognitive deficit.

To help characterise and profile the part 2 participants, this study uses a comprehensive battery of outcome measures at baseline and to assess outcomes following the intervention. One potential uncertainty is whether these measures could be burdensome for participants to complete and therefore affect retention across the three timepoints (baseline, 3-month and 6-month follow-up), particularly amongst our sample of people with MS with cognitive problems. Our initial discussions with PPI members suggest that this will not be the case, but the feasibility and acceptability data gathered throughout the study will help us finalise the outcome measures ahead of the definitive RCT.

The NEuRoMS pathway utilises technological advances including digital administration of cognitive screening, which will afford people with MS the flexibility to complete screening in their own homes, on their own devices, and prior to their clinic appointment. Moreover, results from the cognitive screening will be automatically calculated by an online system and integrated into an easily understandable output made readily available to clinical teams. This could help facilitate conversations about cognitive problems amongst clinicians and people with MS within time-pressured clinical settings. However, some stakeholders may require additional support to use and engage with these digital tools [19, 20]. In addition, clinical services have been differentially affected by the COVID-19 pandemic, adopting different service provision strategies (e.g. a shift to remote delivery/monitoring) and dealing with unpredictable staff absences due to contracting the virus or self-isolation. Such challenges of conducting MS studies during COVID-19 have been described elsewhere [57]. Whilst considerable thought has been given to minimising these challenges, for example by codeveloping the cognitive screening platform with PPI input and working with the three NHS hospitals to embed the NEuRoMS pathway into their different systems, multiple data sources will be gathered to capture the feasibility and acceptability of digital delivery and to address this aspect for a future full-scale trial.

The feasibility study protocol described in this paper forms part of a National Institute for Health and Care Research Programme Grant for Applied Research, titled ‘neuropsychological evaluation and rehabilitation in multiple sclerosis’ (NEuRoMS) project. If feasibility is demonstrated, the definitive RCT will commence in 2023.

## Supplementary Information

**Additional file 1.** SPIRIT 2013 Checklist: Recommended items to address in a clinical trial protocol and related documents*.

## Data Availability

Not applicable

## Abbreviations

AE: Adverse event
CTU: Clinical Trials Unit
DMT: Disease-modifying therapies
GP: General practitioner
ICECAP-A: ICEpop CAPability measure for Adults
MRC: Medical Research Council
MS: Multiple sclerosis
NEuRoMS: Neuropsychological evaluation and rehabilitation in multiple sclerosis
NHS: National Health Service
NWORTH: North Wales Organisation for Randomised Trials in Health
PI: Principal investigator
PPI: Patient and public involvement
RCT: Randomised controlled trial
REC: Research ethics committee
SAE: Serious adverse event
SPIRIT: Standard Protocol Items Recommendations for Interventional Trials
